# Experimental Characterization of Anisotropic Mechanical Behaviour and Failure Mechanisms of Hardened Printed Concrete

**DOI:** 10.3390/ma17163931

**Published:** 2024-08-07

**Authors:** Theresa Glotz, Yuri Petryna

**Affiliations:** Chair of Structural Mechanics, Technische Universität Berlin, Gustav-Meyer-Allee 25, 13355 Berlin, Germany

**Keywords:** 3D printing, additive manufacturing, hardened concrete, mechanical properties, anisotropy, failure mechanisms, experiment, DIC

## Abstract

Extrusion-based printing of cementitious materials represents an innovative technology in civil engineering. The additive manufacturing process significantly influences the material properties in the hardened state, leading to anisotropic behaviour in terms of stiffness and strength compared to conventionally cast concrete. This experimental study aims to deepen the understanding of the mechanical behaviour of hardened printed concrete. Beam-like specimens with varying printing patterns, loading orientations and lengths are investigated within three-point bending tests (3PBT) and uniaxial compression tests (UCT). Homogenized material parameters such as Young’s modulus, compressive and flexural tensile strength and density are statistically evaluated using optically measured displacement and strain fields on the specimen surface. The qualitative and quantitative results demonstrate a strong dependency of material properties and failure mechanisms on the printing pattern. The interfilamental and interlayer areas with weak adhesion are identified as the main reason for anisotropy.

## 1. Introduction

The adoption of 3D printing processes in the construction industry is transforming the building sector and expanding perspectives on construction methods. The contour crafting approach introduced by Khoshnevis [1,2] marked a significant milestone, paving the way for subsequent developments in additive manufactured concrete, as summarized in [3]. 3D concrete printing (3DCP) enables the creation of complex structures with high design flexibility, enhanced safety standard by reducing physical labour, improved material efficiency and cost reductions [3,4,5,6]. An overview of the different additive manufacturing techniques can be gained in [6,7]. The research field for extrusion-based 3DCP is vast, with various aspects requiring focused study. In the fresh state, materials must be pumpable, extrudable, printable and capable of meeting buildability requirements [6,8]. Consequently, significant emphasis is placed on material mix design in 3DCP research. Further research areas include the integration of reinforcement and digital aspects such as process control and modelling of printed concrete and related processes [9,10].

However, the properties and performance of the hardened printed components are decisive for the practical application. Unlike conventionally cast concrete, the layer-by-layer deposition process of 3DCP results in anisotropic material behaviour, which significantly influences the mechanical performance and emerges as a critical mechanical property [11,12,13]. This anisotropy has been investigated in numerous studies. Ding et al. provide a critical overview of recent research focused on interfaces in printed concrete. From a microstructural perspective, the primary influencing factors include interface humidity, moisture transport processes and the characteristics of the microporous structure [11].

Anisotropy in printed concrete is evident at multiple levels. Wolfs et al. identify the interlayer interval time as a significant factor influencing bond strength [14]. Other studies also report that the mechanical performance depends on the adhesion between printed layers, with interface properties deteriorating as interval times increase [12,15]. Similarly, Sanjayan et al. found that the print-time interval affects interlayer strength and correlated this to the surface moisture content at the interface of the layers [16].

Investigations focus not only on the presence of interlayers but also on their orientation concerning the applied load. The mechanical properties exhibit anisotropic character depending on the orientation of the layers and the direction of load application [17]. Ding et al. showed the effect of orientation on tensile splitting strength through experimental investigations with recycled sand [18]. Additionally, Kumar et al. reported that in a test series with translational and depositional interfaces, the compressive strength of printed concrete was lower, but the flexural strength was higher compared to cast specimens [19].

From a micro-structural perspective, several investigations have demonstrated higher local porosity in the interlayers by analyzing the air void content in both the bulk material and interlayer regions [20,21]. The presence of macro pores is a determining factor for weak interface adhesion [17]. Liu et al. analyzed the effect of pore structure on printed concrete with coarse aggregate, establishing a connection between pore defect geometry and cracking damage [22]. Some studies have advanced further by applying cementitious paste at the interface to increase bond strength, a technique successfully demonstrated by [23].

The analysis of fracture mechanisms due to the additive manufacturing process is addressed in only a few studies [24]. Crack propagation in specimens has been shown to occur predominantly along the interface of two layers during splitting prism tests [25]. An examination of layer height effects on the flexural and fracture response of plain and fiber-reinforced 3D-printed beams indicated that smaller layer heights offer benefits, despite introducing more interfaces and requiring longer printing times [26]. Some studies have included numerical simulations using finite element (FE) modelling strategies to predict structural performance in the hardened state and the failure mechanisms of reinforced concrete beams under various loading conditions, showing good agreement with experimental results [27,28]. Pi et al. investigated the crack propagation and failure mechanisms of specimens reinforced with 2% polyvinyl alcohol fibers with a length of 12 mm. Under bending loads, two new crack propagation modes were identified: bending cracks leading to the splitting of adjacent filaments and localized shear cracks that dominate in cases of excessively low interlayer bond strength [29]. In [30], the acoustic emission technique was used to monitor the micro-cracking mechanism in printed concrete. The most recent study by Tang et al. distinguishes between two different crack types depending on the propagation mode in notched three-point bending tests: trans-layer and inter-layer fractures [24].

Despite the extensive research conducted so far, there is a remaining need for further investigation into certain aspects of the mechanical performance of printed concrete. Most experimental studies use specimens that have been printed and then saw-cut for testing. While this approach facilitates comparative analysis, it is limited by the lack of unified test methods and standards, which complicates the comparison of results from different studies [11,31]. Mechtcherine et al. have noted that by cutting the sides of the outer regions, the effects on mechanical performance can no longer be accurately assessed [32]. Furthermore, there is a need for a more thorough investigation of the fracture mechanisms of the entire component.

This study aims to address these gaps by investigating a series of beam-like printed concrete specimens with different printing patterns in three-point bending tests (3PBT) with varying loading orientations and uniaxial compression tests (UCT) with different specimen lengths. The specimens feature multiple filaments per layer, introducing not only interlayer but also interfilament interfaces. UCT is conducted on whole specimens rather than cubes to better observe failure modes. Digital image correlation (DIC) techniques are used for optical measurements, as applied in previous studies [18,24,26,33,34]. For both tests, the results are evaluated with respect to mechanical parameters such as stiffness, strength and density. Additionally, the failure mechanisms are analyzed in detail to gain a comprehensive understanding of the mechanical processes in the material at a macroscopic scale, without the influence of reinforcement. This research seeks to gain a deeper understanding of the structural performance of printed concrete, taking into account the actual printed cross-sectional geometry that varies due to the additive manufacturing process.

## 2. Materials and Methods

### 2.1. Printing System and Concrete Mix Design

The printer used to manufacture the specimens is a xyz-gantry system incorporating an active pumping nozzle for material deposition designed at TU Berlin, for further information see [35]. The material employed for the printing process is a cement-based mortar consisting of 900 g CEM III/A 42.5 N, 600 g aggregates (⌀ 0.1 mm to 0.6 mm), 470 g powdered limestone, 485 g water, 9 g defoaming agent (SIKA Control 300 PerFin) and 4 g short basalt fibers of 6 mm length. A detailed investigation of the material composition referred to as G0 was carried out by Cuevas et al. in [36], and it was also applied in [37].

### 2.2. Test Series

**Figure 1:**
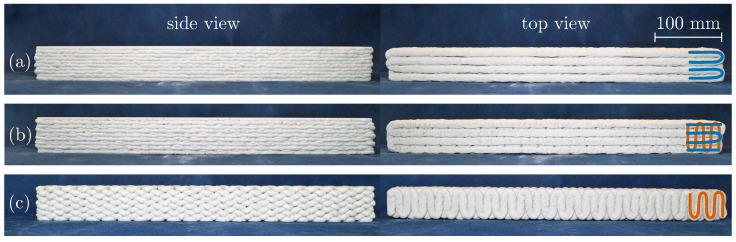
Overview of printed specimens in side and top view with different printing patterns: (**a**) lengthwise (L), (**b**) alternately length-/crosswise (LC) and (**c**) crosswise (C) printed filaments.

In order to systematically investigate the anisotropic properties of the material in longitudinal and transverse direction, three different printing patterns are applied with lengthwise (L), alternately length- and crosswise (LC) and crosswise (C) printed filaments, see Figure 1a–c. For LC specimens, this means that the filaments of the first layer are printed in the lengthwise direction, the second layer in the crosswise direction, the third layer again in the lengthwise direction, and so on. The printing time for a single L specimen is approximately 20 min, whereas the printing time for LC and C specimens is increased by 10% and 15% respectively, due to the higher number of directional changes in the printing path for crosswise printed filaments. In total, 30 beam-like specimens with a desired geometry of 50×50×500 mm are printed, with ten specimens for each of the three printing patterns. The primary objective is to test the specimens in their original geometry. Nevertheless, some specimens need to be saw-cut to achieve a plain load application surface.

### 2.3. Experimental Setup

**Figure 2:**
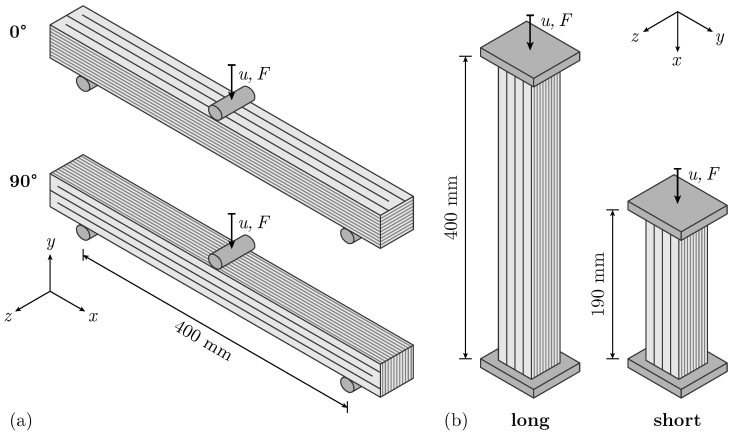
Overview of experimental test setup: (**a**) Displacement-controlled three-point bending test (3PBT) with 0°- and 90°-oriented specimens, (**b**) displacement-controlled uniaxial compression test (UCT) with long and short specimens.

The experimental testing arrangement consists of two parts. The first part involves a 3PBT conducted until failure, with a support span of 400 mm using two different combinations of layer orientation and loading direction, see Figure 2a. A 0° orientation describes a loading direction perpendicular to the printing path direction, whereas a 90° orientation indicates load application to the perpendicular edge of the beam. The specimens tested with 90° orientation are saw-cut along their long side to obtain even surfaces at the support and load application points.

As a second part, a UCT with two different specimen length is conducted in order to investigate the influence of the specimen length on the mechanical performance (Figure 2b). The long and short specimens are saw-cut to a length of 400 mm and 190 mm respectively, with minor deviations due to the sawing process. As shown in Figure 2, the *x*-axis of the applied coordinate system is always aligned with the longitudinal axis of the specimen for both 3PBT and UCT. The *x*-*y*-plane spans the surface for the optical displacement and strain measurements.

All test are conducted under displacement control with rates of 100 μm/min and 250 μm/min (3PBT) and 500 μm/min (UCT). The applied machine force is continuously monitored for the induced displacement at the midpoint of the specimen. Additionally, the displacement and strain fields on the front side of the specimen are continuously measured (see Section 2.4.2).

Table 1 and Table 2 show an overview of the performed tests for each configuration. Some specimens could not be tested due to defects, leading to the shown number of specimens. Due to their poor performance in terms of interfilamental bond strength in 3PBT, C specimens are excluded after the first 3PBT_C. All remaining C specimens are tested under compressional load, resulting in a higher number of specimens for UCT_C (see Table 2).

### 2.4. Optical Measurements

#### 2.4.1. Geometric Surveying by Structured Light Scanning

**Figure 3:**
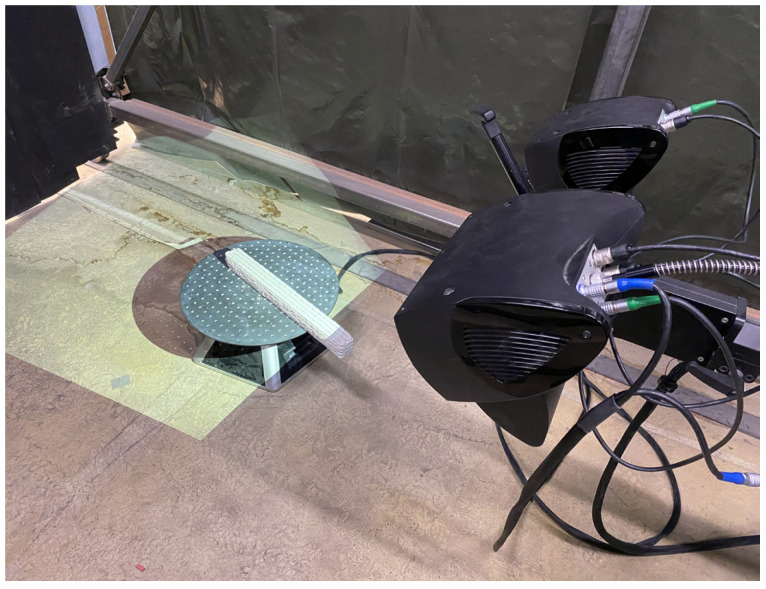
Structured light scanning system COMET 5 with specimen on rotary table used for geometric all-round surveying.

The additive manufacturing process entails deviations of the manufactured specimen geometry from the target geometry. The actual printed geometry is consciously taken into account in the present test series. The optical structured light scanning system COMET 5 by Steinbichler Optotechnik GmbH, Neubeuern, Germany (now Carl Zeiss Optotechnik GmbH), is employed to capture the specimen’s precise surface geometry prior to testing in order to quantify deviations (Figure 3). The system projects a series of stripe patterns onto the specimen’s surface and subsequently calculates 3D surface coordinates from the intersections of the stripe patterns with the camera grid using the principle of triangulation. For further information on the operating principle, see [38].

#### 2.4.2. Optical Deformation Measurements

**Figure 4:**
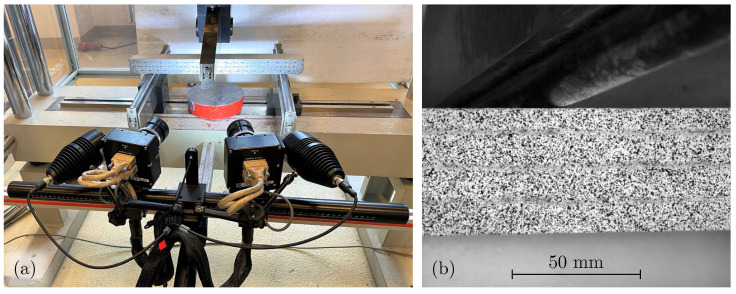
(**a**) Three-point bending test setup with ARAMIS 4M optical stereo camera system. (**b**) Sprayed stochastic grey value pattern on the specimen surface.

During the test, the deformations on the specimen’s visible surface are measured using the optical stereo camera system ARAMIS 4M manufactured by GOM GmbH, Braunschweig, Germany (now Carl Zeiss Industrial Quality Solutions GmbH; Figure 4a). The system operates contactless with one sensor and two cameras and is able to capture 3D coordinates, 3D displacements and surface strains. The measurement method of the sensor is based on the principle of DIC. The recognition of image areas, referred to as facets, in both the left and right camera enables the process of DIC [39]. Therefore, the specimen’s surface is prepared with a thin sprayed random grey value pattern that doesn’t influence the mechanical behaviour (see Figure 4b).

The measurement accuracy, which varies with the measurement volume, achieves a precision of 0.01 mm for the current configuration. For 3PBT, the image generation sampling frequency is set at $f_{s}$ = 2 Hz to 4 Hz. In UCT, it is possible to generate images with $f_{s}$ = 2 Hz while utilizing a ring memory to store the last 100 images at a higher frequency of $f_{s}$ = 20 Hz due to a system upgrade.

## 3. Results

### 3.1. Three-Point Bending Test

#### 3.1.1. Failure Mechanisms

**Figure 5:**
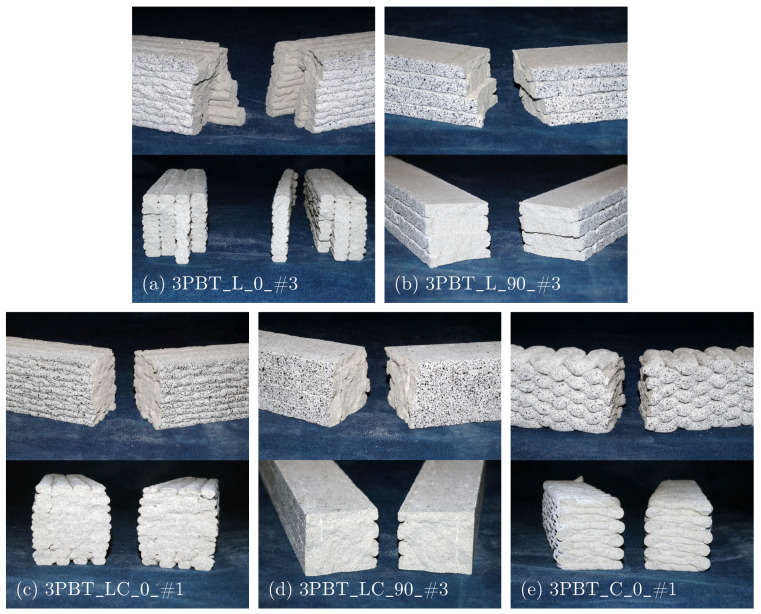
Fracture surfaces of 3PBT: lengthwise printed specimens with (**a**) 0° and (**b**) 90° orientation, length-/crosswise printed specimens with (**c**) 0° and (**d**) 90° orientation and (**e**) crosswise printed specimen with 0° orientation.

As expected, the beam-like unreinforced conrete specimens fail in 3PBT due to a discrete crack in the beam centre beneath the load application point. The brittle fracture surfaces of 3PBT are shown in Figure 5. The filaments of L_0 specimens behave like four individual planes (Figure 5a). The very low interfilamental bond even leads to a separation of a filamental plane. The cracks develop in each filament row individually.

Similarly, the crack evolves gradually over the specimen height in L_90 specimens (Figure 5b). LC specimens behave more homogeneously both for 0° and 90° orientation (Figure 5c,d). A sharp edge on the fracture surface of Specimen 3PBT_LC_90_#3 can be detected as an indication for an interfilamental gap between crosswise printed filaments (Figure 5d). The fracture surface of the only 3PBT_C specimen in Figure 5e reveals just a selective filamental bond at the location of directional changes in the printing path, explaining the poor performance in 3PBT.

**Figure 6:**
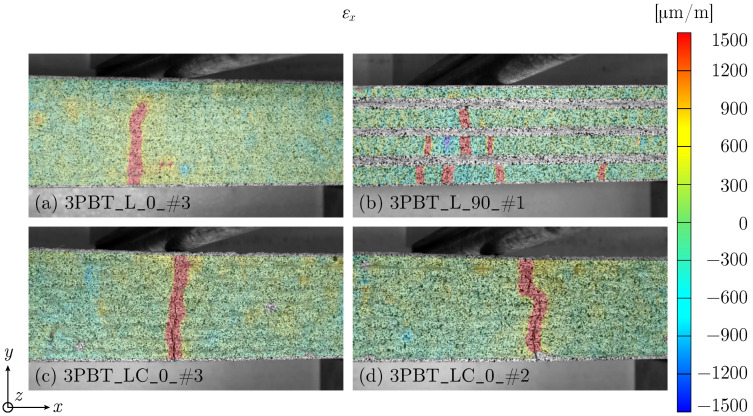
Strain field $\varepsilon_{x}$ at failure: (**a**) 3PBT_L_0_#3, (**b**) 3PBT_L_90_#1, (**c**) 3PBT_LC_0_#3 and (**d**) 3PBT_LC_0_#2.

The evaluation of the measured strain field for 0°-oriented specimens shows mainly crack patterns with one dominant crack leading to failure, independent of the printing pattern (Figure 6a,c,d). The crack does not always necessarily initiate in the beam middle, as demonstrated in Figure 6d.

**Figure 7:**
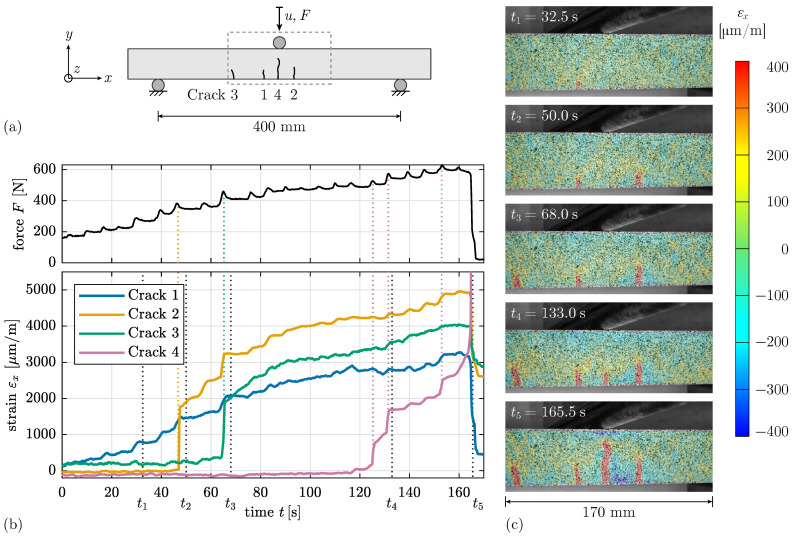
(**a**) Schematic illustration of displacement-controlled 3PBT for Specimen 3PBT_LC_90_#3 with order of crack appearance. (**b**) Evolution of strain $\varepsilon_{x}$ at location of Cracks 1 to 4 at the bottom edge of the specimen and machine force *F* over time *t*. (**c**) Strain field $\varepsilon_{x}$ with crack evolution for specific time points $t_{1}$ to $t_{5}$.

In contrast to one crack, 90°-oriented L and LC specimens show a crack pattern with formation of multiple cracks, see Figure 6b and Figure 7. The gradually developing crack observed for L_90 specimens from the fracture surface (Figure 5b) can also be seen in the strain data in Figure 6b.

The optical measurement data allows a detailed analysis of the failure mechanism of Specimen 3PBT_LC_90_#3 over time in Figure 7. The strain data $\varepsilon_{x}$ at the location of each crack obtained at the bottom edge of the specimen shows a continuous increase of strain for Crack 1, whereas the strain development in Cracks 2 and 3 is dominated by a sudden increase at specific time points. Crack 4 in the beam centre leads to failure (Figure 7a,b). Figure 7c shows the strain field for different time points $t_{1}$ to $t_{5}$ right after the strain in the cracks increases. An increasing strain in Crack 4 is accompanied by decreasing strains in the other cracks.

It can be observed that the machine force correlates with the crack appearance and evolution. A slight increase and subsequent decrease in the load occurs simultaneously with the increase in strain within the cracks (indicated by dashed coloured lines in Figure 7b). After a crack opening, the stresses redistribute and the load can be increased further. The other peaks in the force curve suggest the formation of additional cracks in other areas of the specimen, not visible on the measured surface.

#### 3.1.2. Material Properties

The effect of anisotropy can be made measurable by applying the assumption of homogeneous linear-elastic material behaviour to determine the material properties from measured load, displacement and strain values. To calculate the Young’s modulus from 3PBT, the displacement at the load application point on the top edge of the beam centre is determined from the optical measurements. Subsequently, the Young’s modulus can be calculated using linear beam theory:

(1) $$E=\frac{\Delta F\;\cdot \;L^{3}}{\Delta u\;\cdot \;48I}\;,$$

with moment of inertia $I=\frac{b\;\cdot \;h^{3}}{12}$, support span *L* and incremental values for force ΔF and displacement Δu. The latter can be approximated using a linear fit of the force-displacement relationship, which is linear until reaching the ultimate load due to tensile failure. The cross-sectional information for height *h* and width *b* are measured individually for each specimen.

The brittle failure in 3PBT occurs due to a rapid growth of the central crack when the tensile stress on the bottom side of a specimen exceeds the tensile strength. The ultimate load at failure is not explicitly shown due to varying cross-sections, but its normalised value correlates with the flexural tensile strength $f_{t}$ (also referred to as flexural strength, e.g., [18,21,24]) that is calculated using

(2) $$f_{t}=\frac{F\;\cdot \;\frac{L}{4}}{\frac{b\;\cdot \;h^{2}}{6}}=\frac{3\;\cdot \;FL}{2\;\cdot \;bh^{2}}\;,$$

where *F* is the recorded ultimate load.

**Figure 8:**
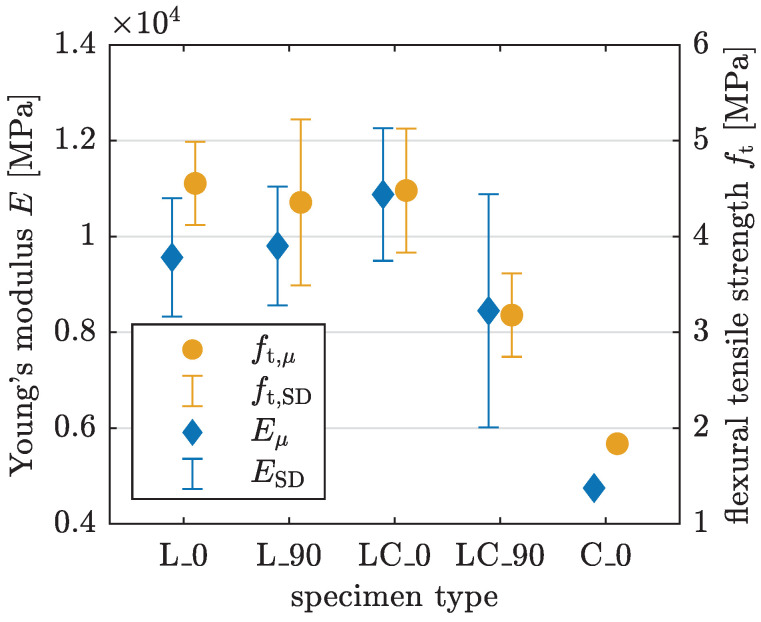
Young’s modulus *E* and flexural tensile strength $f_{t}$ derived from 3PBT with mean value μ and standard deviation SD.

The results are shown quantitatively in Table 3 and are visualized in Figure 8. For L specimens, the orientation does not significantly affect the material parameters. In contrast, LC specimens exhibit a 22% lower mean value for the Young’s modulus and a 29% lower mean value for the flexural tensile strength when oriented at 90°. However, the standard deviation for LC_90 specimens is higher. The material parameters for the C_0 specimen confirm the already observed underperforming of the C pattern in 3PBT. Table 3 shows homogenized material properties for the printed concrete which are significantly lower than those of cast concrete and exhibit differences conditioned by the applied printing pattern.

### 3.2. Uniaxial Compression Test

#### 3.2.1. Failure Mechanisms

**Figure 9:**
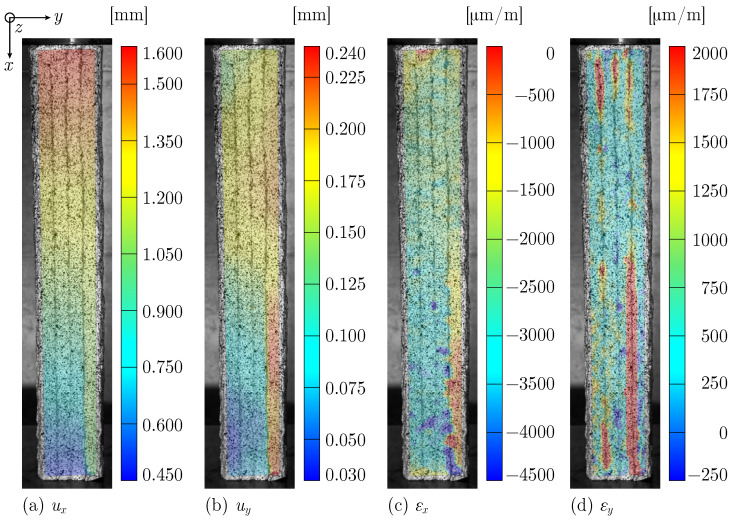
Measured displacement fields (**a**) $u_{x}$ and (**b**) $u_{y}$ and strain fields (**c**) $\varepsilon_{x}$ and (**d**) $\varepsilon_{y}$ just before failure for Specimen UCT_L_l_#1.

The printed specimens exhibit quite different failure mechanisms in UCT and therefore require individual investigation. The failure behaviour of L specimens under compression is characterized by the detachment of filaments. The optical measurement data for Specimen UCT_L_l_#1, shown in Figure 9a–c, reveals increasing vertical and horizontal displacements $u_{x}$ and $u_{y}$ and strains $\varepsilon_{x}$ for the right filament compared to the other three filaments. The strain component in *y*-direction (Figure 9d) further illustrates the delamination, with increased strain values at the filamental boundaries.

**Figure 10:**
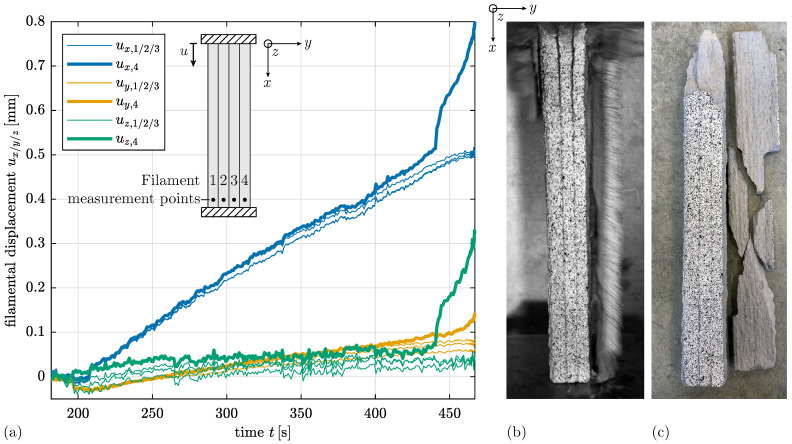
(**a**) Evolution of displacement *u* in *x*-,*y*- and *z*-direction measured at the bottom of each Filament 1 to 4 over time *t* for Specimen UCT_L_l_#1. (**b**) Failure mechanism with detachment of right filament at the moment of failure captured from optical measurements. (**c**) Broken specimen.

An extended evaluation of the optical measurement data provides a deeper understanding of the mechanical behaviour. Figure 10a displays the displacement of each of the four filaments at the bottom of Specimen UCT_L_l_#1 in the three spatial directions over time. The displacement in *x*-direction parallel to the loading direction dominates. Beginning at t≈440 s, the displacement values for Filament 4 start to diverge from those of the other three filaments. The *z*-displacement indicates that Filament 4 moves forward while its vertical displacement increases significantly more than that of the other three filaments. This implies that Filament 4 bears a greater load and consequently fails earlier under compressive load due to lateral deflection. Not only does Filament 4 detach, but Filaments 1 to 3 also detach in the area of load application, as shown in Figure 10b. Figure 10c illustrates the broken specimen with the completely detached filament and the upper fractured part.

**Figure 11:**
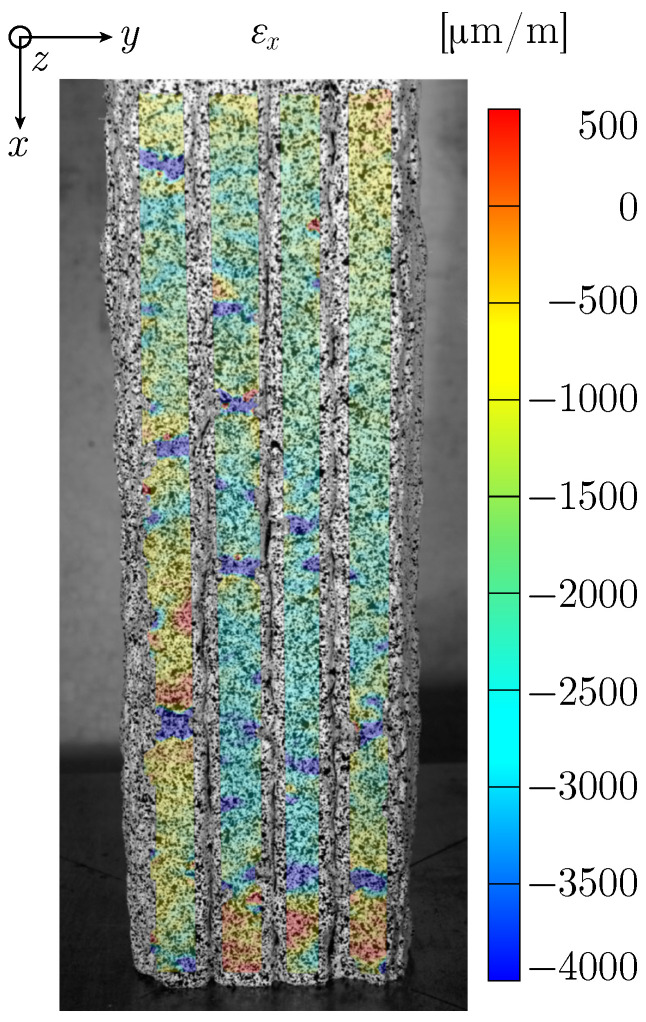
Strain field $\varepsilon_{x}$ for Specimen UCT_L_s_#2 just before failure.

The evaluation of the strain field data for Specimen UCT_L_s_#2 enables the detection of localized weak points that are characterised by increased compression, see Figure 11. These areas arise due to a fluctuating extrusion rate of the material from the printing nozzle.

**Figure 12:**
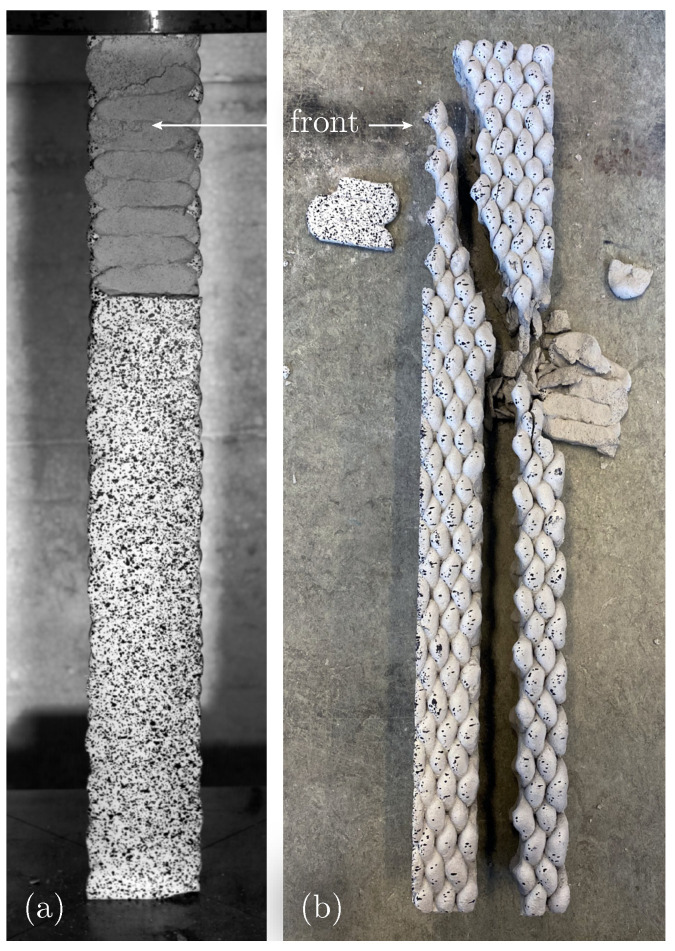
Failure mechanism of Specimen UCT_C_l_#1: (**a**) front view with detached upper part of front layer, (**b**) broken specimen in side view.

**Figure 13:**
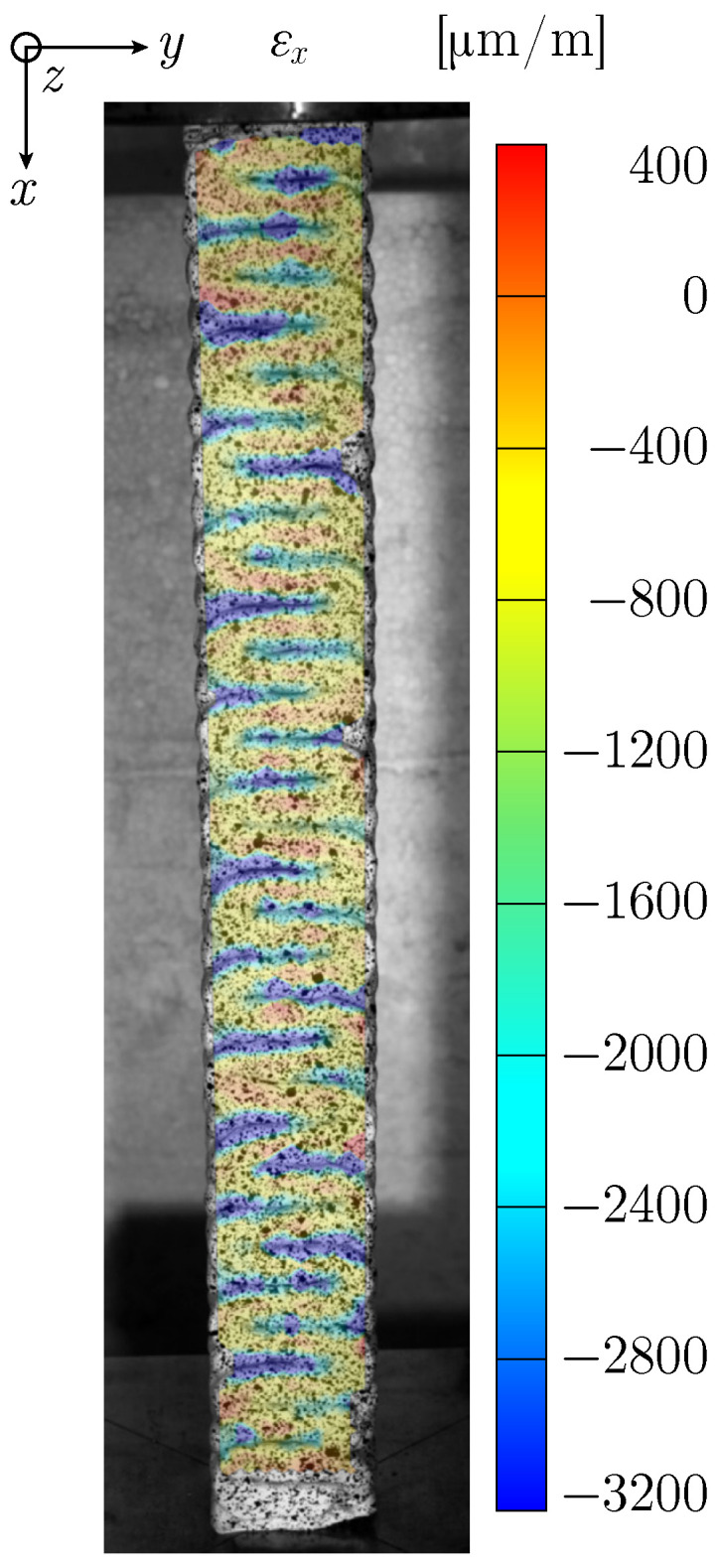
Strain field $\varepsilon_{x}$ for Specimen UCT_C_l_#4 just before failure.

In long C specimens under compression, two types of cracks appear: slanted cracks originating from the top, caused by the constraint of lateral expansion at the load application plate, and vertical cracks along the layer boundary (Figure 12b). For Specimen UCT_C_l_#1, detachment of the upper part of the front layer is observed (Figure 12a). The increased negative strain data in Figure 13 indicate compression of the interfilamental gaps resulting from the crosswise printing process. The significant compression in these regions leads to a lateral expansion, causing fractures at points of weak adhesion.

**Figure 14:**
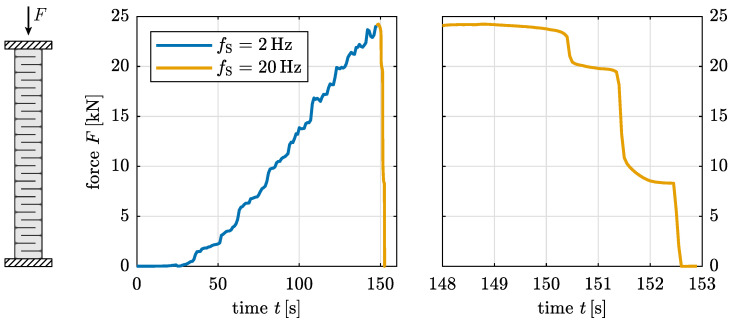
Applied machine force *F* over time *t* with detailed view of post-fracture range measured with sampling frequency $f_{s}$ = 20 Hz for Specimen UCT_C_l_#4.

The displacement-controlled test setup allows a further insight into the failure mechanism once the ultimate load is reached. For C specimens, the measured load gradually decreases after exceeding the ultimate load due to the formation of new cracks (see Figure 14 for Specimen UCT_C_l_#4). As the cracks propagate, the load-bearing cross-section diminishes, leading to a gradual reduction in load capacity with each new crack. This observation further emphasises the anisotropic structure of the material.

**Figure 15:**
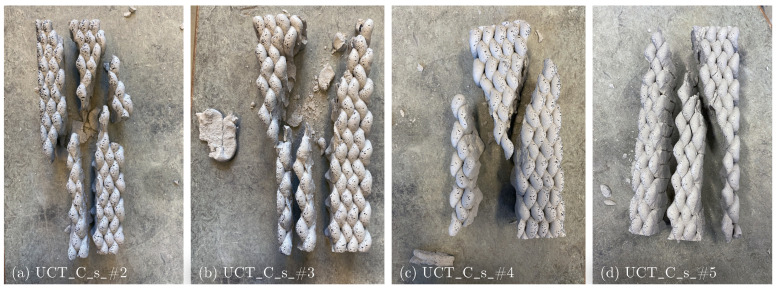
Failure mechanism of short crosswise printed specimens: (**a**) UCT_C_s_#2, (**b**) UCT_C_s_#3, (**c**) UCT_C_s_#4 and (**d**) UCT_C_s_#5.

Short C specimens exhibit similar crack patterns, with shear cracks originating at the top and propagating along the filamental printing boundary (Figure 15a–d). In the lower half, the crack propagates vertically along the layer boundary, parallel to the loading direction. Additionally, specimens #2, #3 and #5 display multiple cracks parallel to the loading direction (Figure 15a,b,d). The C printing pattern is characterized by a higher number of directional changes in the printing path and therefore a longer interlayer interval time, leading to reduced adhesion between both filaments and layers.

**Figure 16:**
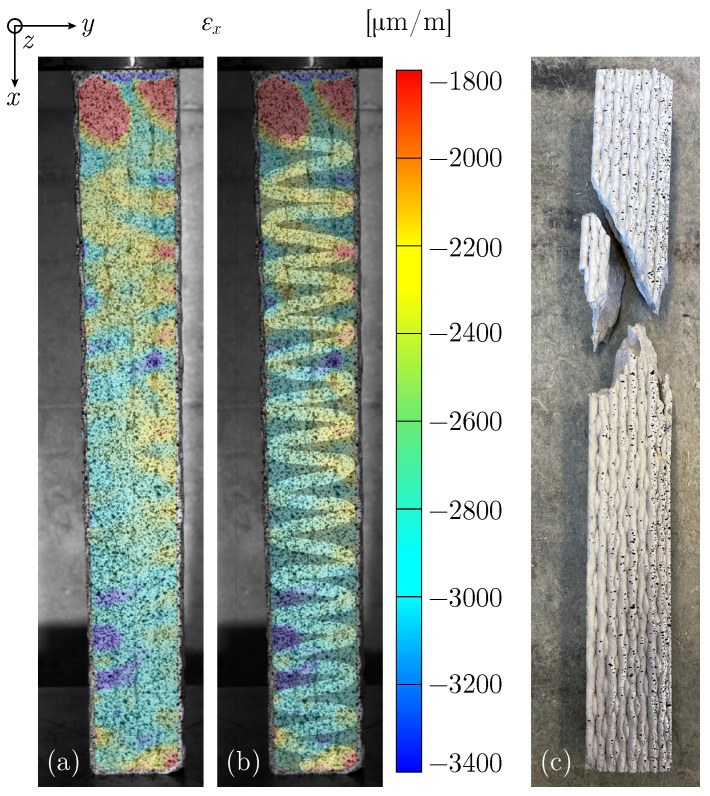
Specimen UCT_LC_l_#2: (**a**) Strain field $\varepsilon_{x}$ just before failure with (**b**) highlighted regions. (**c**) Broken specimen.

For LC specimens, the boundaries between lengthwise and crosswise printed filaments are visible in the strain field distribution (see Figure 16a). Increased strain values on the visible surface layer with lengthwise printed filaments follow a pattern that orients on the underlying second layer printed with crosswise filaments. Figure 16b highlights the trajectory of the underlying crosswise printed filaments, where improved adhesion results in lower compression. The porous space between crosswise printed filaments at directional changes in the printing path leads to a higher compression and therefore increased strains under compressive load.

LC specimens perform significantly better in maintaining interfilamental and interlayer connections. The failure mechanisms of both long and short LC specimens are predominantly similar to those for conventionally cast concrete, as shown in Figure 16c.

#### 3.2.2. Material Properties

**Figure 17:**
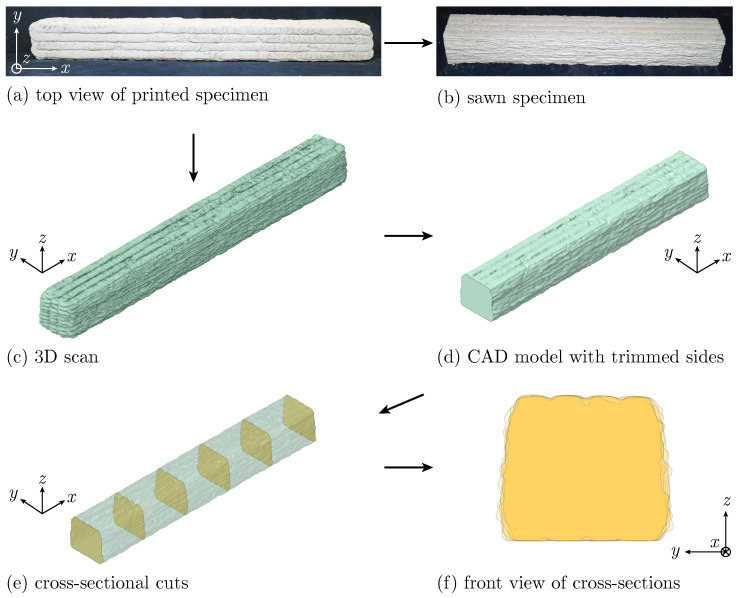
Post-processing scheme of 3D surface scans of specimens to obtain the cross-sectional area and volume information, exemplary shown for Specimen UCT_L_l_#1.

Figure 17 schematically illustrates how the 3D scan of the specimens, conducted prior to testing, is utilized to obtain geometric information. The specimen’s surface (Figure 17a) undergoes structured light scanning (Figure 17c). Since UCT specimens are cut to ensure an even load application area (Figure 17b), the CAD model is similarly trimmed to match this length (Figure 17d). Data from six cross-sectional cuts along the specimen’s length (Figure 17e) are processed to derive mean values for each specimen’s cross-sectional area. Variations in cross-sectional shapes are depicted in Figure 17f, presented in an overlaid front view. Additionally, the CAD model is used to calculate the volume of the specimens.

**Figure 18:**
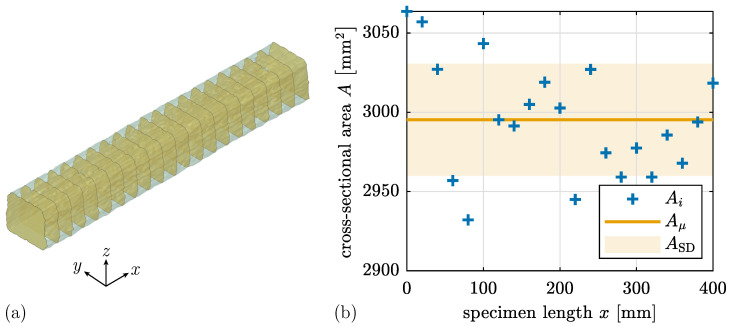
(**a**) Visualization of cross-sectional cuts in CAD model. (**b**) Discrete values of cross-sectional area $A_{i}$, mean value $A_{\mu }$ and standard deviation $A_{\mathrm{SD}}$ over specimen length *x* for Specimen UCT_L_l_#1.

The variation of the cross-section along the specimen’s length is exemplary investigated on Specimen UCT_L_l_#1, see Figure 18a. Therefore, twenty-one cross-sections are individually evaluated from the scanned geometry. The cross-sectional area varies, although only with minor deviations from the mean value (Figure 18b). The economic compromise involves using six cuts while still achieving satisfactory results.

**Figure 19:**
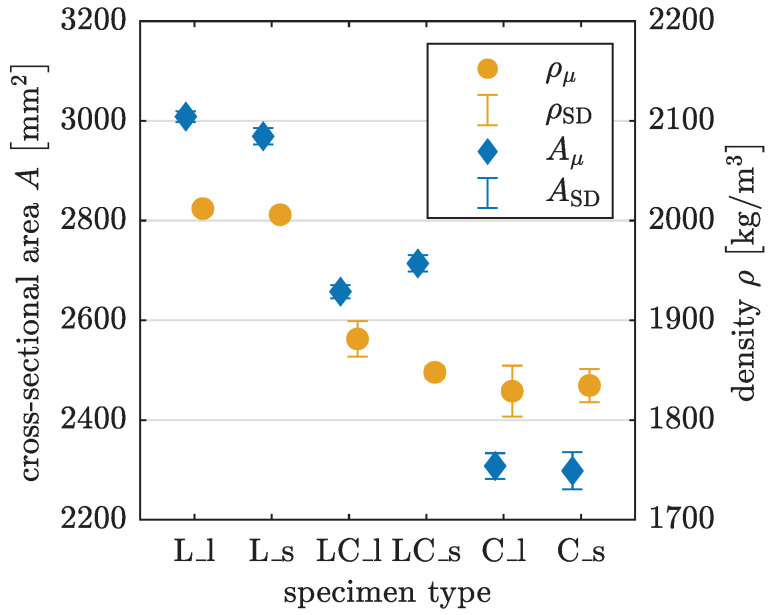
Cross-sectional area *A* and density ρ for UCT specimens with mean value μ and standard deviation SD.

The coefficient of variation for the cross-sectional area *A* among all UCT specimens is less than 1.6%, indicating relatively low variability in area (see Table 4 and visualized in Figure 19). However, the area deviates from the target geometry of

(3) $$A_{\mathrm{target}}=50\;\mathrm{mm}\cdot 50\;\mathrm{mm}=2500\;{\mathrm{mm}}^{2},$$

see Table 4. The cross-sectional area for L specimens exceeds the target value by around 20%, while for C specimens, it is 8% lower. Therefore, considering the actual geometric dimensions has a relevant impact on the results.

Since the specimens are also weighted before testing, the density ρ can be determined using the mass *m* and the volume *V* obtained from the 3D scan:

(4) $$\rho =\frac{m}{V}\;.$$

The calculated densities in Table 4 represent values that include all interfilamental and interlayer voids resulting from the printing process. LC and C specimens achieve approximately 92% of the mean density value calculated for L specimens, indicating that L specimens are printed in a more compact manner.

Based on the acquired cross-sectional area data, the stress in the specimen is subsequently calculated by

(5) $$\sigma =\frac{F}{A}\;,$$

where *F* represents the recorded machine force and *A* the mean value of the cross-sectional area obtained for each specimen from structured light scanning. With the surface strains determined by optical measurements it is possible to determine an integral Young’s modulus *E* via

(6) $$E=\frac{\sigma }{\varepsilon }\;,$$

where *E* is computed using a linear fit of the stress-strain curve within the initial linear range. Besides, the maximum measured stress $\sigma_{\max}$ can be considered as the compressive strength $f_{c}$:

(7) $$f_{c}=\sigma_{\max}\;.$$

**Figure 20:**
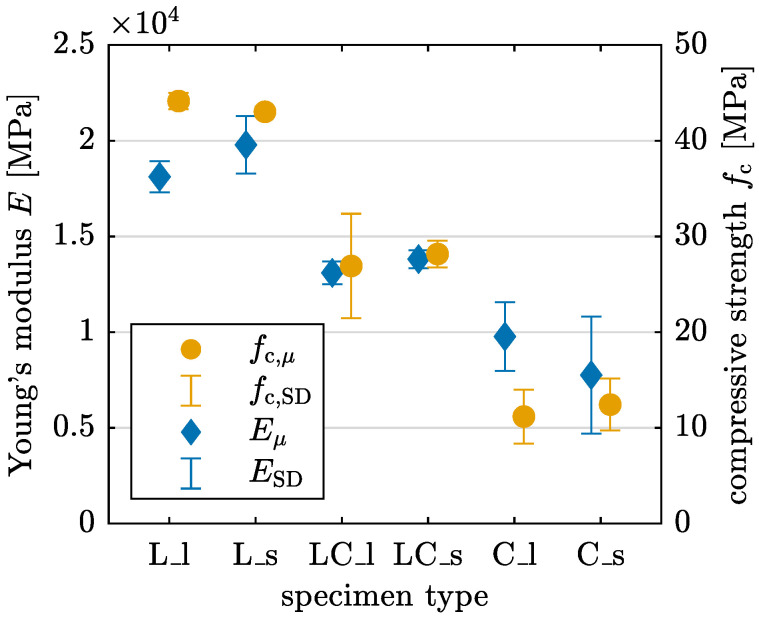
Young’s modulus *E* and compressive strength $f_{c}$ derived from UCT with mean value μ and standard deviation SD.

Table 5 and Figure 20 show the Young’s modulus and the compressive strength for each combination of printing pattern and specimen length. L specimens have the highest Young’s modulus, while C specimens show the lowest one with significant differences in the magnitude. The same trend is observed in compressive strength. The Young’s modulus for short specimens is slightly higher than for long specimens, except for C specimens. However, short specimens C_s have a notably high coefficient of variation at 39.4%. The difference in compressive strength between short and long specimens for each printing pattern is relatively small.

## 4. Discussion

The experimental investigation reveals significant differences in failure mechanisms and mechanical properties among the tested specimens. The variation of the printing path in longitudinal and crosswise direction allows a systematic evaluation regarding different combinations of printing path and loading direction.

The first observation relates to the failure mechanisms. Fracture surfaces and optical measurements from 3PBT indicate that the failure mechanism of the specimens depend on the orientation of the printed layers. Specimens with a 0° orientation tend to fail with one dominant crack, whereas those with a 90° orientation show a pattern with multiple cracks. The interfilamental region is particularly critical. For instance, in LC_90 specimens, the results suggest that crack formation occurs at weak connection points of crosswise printed filaments. This affects the spatial stiffness distribution, which adapts over time as new cracks appear.

The failure mechanisms for UCT vary significantly depending on the printing pattern. For L specimens, the failure mode is primarily characterized by the detachment of filaments, which may be influenced by uneven load distribution or imperfections in load application or geometry. In contrast, for LC and C specimens, gaps at the location of directional changes in the printing path in crosswise printed filaments create weak points that become more compressed during testing. The interfilamental and interlayer bonds are critical factors for C specimens, as evident in the fracture surfaces of both long and short specimens. Among the three investigated printing patterns, LC specimens exhibit the most homogeneous behaviour. The results obtained regarding crack propagation along the interface of two layers in compression tests are consistent with observations made by kZareiyan and Khoshnevis in [25] for some of their specimens, despite differences in the experimental setup.

The calculated material parameters yield the following observations: Within the same test setup, L specimens exhibit the best performance with respect to flexural tensile strength in 3PBT and regarding compressive strength and Young’s modulus in UCT, implying superior load-bearing capacity. Conversely, C specimens demonstrate the poorest performance across all disciplines. The two effects leading to the results for C specimens are an increased interlayer interval time, which influences the interlayer bond, as shown in [12,14,15], and the geometrical configuration, which influences the interfilamental bond. The experimental results underline that the latter has a greater influence in the present test series. The different specimen lengths tested do not significantly influence the results. The increased variation in the resulting parameters indicates that control of the printing process is essential for generating replicable results. For 3PBT, the highest Young’s modulus is observed in LC_0 specimens. However, the absolute values must be interpreted with caution, as there is a significant discrepancy between the Young’s modulus results obtained from 3PBT and UCT. This discrepancy indicates that calculating an averaged value for the entire specimen based on linear beam theory or a homogenized compression member is insufficient for determining accurate material parameters.

The investigation of the actual specimen geometry using 3D structured light scanned surface data demonstrates that the actual geometry differs for the three different printing patterns and also does not meet the targeted dimensions. Accounting for the measured cross-sectional area significantly impacts the resulting material parameters.

## 5. Conclusions

The findings of this research contribute to a deeper understanding of the mechanical performance of additively manufactured concrete. The investigation of failure mechanisms and material properties of beam-like printed specimens with various printing patterns-lengthwise, alternately length- and crosswise and crosswise printed filaments-reveals distinct performance characteristics.

The study confirms the anisotropic behaviour of printed concrete. Furthermore, it provides profound insights into failure mechanisms under bending and compression. Specimens oriented at 90° exhibit a multiple crack pattern under bending load, contrasting with 0°-oriented specimens dominated by a single crack. The interfilamental bond emerges as a critical factor influencing failure behaviour. Weak points can lead to inducement of cracks that are not necessarily originating at points of maximum stress. Under compression, failure mechanisms vary significantly depending on the printing pattern. While the failure mechanism for L specimens is characterized by filament detachment, for C specimens it is dominated by cracks propagation along interfilamental and interlayer boundaries. Only the failure mode of LC specimens is comparable to that of cast concrete. Standardized cubic geometries that are typically used for determining compressive strength parameters would not allow for the observation of these structural behaviours.

The obtained material parameters, such as Young’s modulus, compressive and flexural tensile strength and density, highlight pattern-dependent mechanical properties. This is particularly problematic for C specimens due to a low interlayer bond observed in UCT and a weak interfilamental bond manifested in 3PBT. This printing pattern is critical for achieving optimal structural performance.

Understanding the actual specimen geometry using 3D structured light scanned surface data underscores its crucial role in accurate assessment. While research methods that saw-cut specimens from objects are important for determining material parameters, they do not capture the true printed geometry.

For further research, such as FE modelling of printed concrete, this study offers essential insights, emphasizing the need for a comprehensive approach to account for direction-dependent mechanical behavior in printed concrete. 

## Figures and Tables

**Table 1:** Overview of specimens for 3PBT.

Test	Printing Pattern	Orientation	Number of Specimens
Three-point bending test (3PBT)	Lengthwise (L)	0°	4
		90°	3
	Length-/crosswise (LC)	0°	3
		90°	3
	Crosswise (C)	0°	1
		90°	-

**Table 2:** Overview of specimens for UCT.

Test	Printing Pattern	Specimen Length	Number of Specimens
Uniaxial compression test (UCT)	Lengthwise (L)	Long (l)	2
		Short (s)	2
	Length-/crosswise (LC)	l	3
		s	2
	Crosswise (C)	l	4
		s	6

**Table 3:** Young’s modulus *E* and flexural tensile strength $f_{t}$ derived from 3PBT with mean value μ, standard deviation SD and coefficient of variation CV.

Printing Pattern	Young’s Modulus *E*			Flexural Tensile Strength $f_{t}$		
	μ [MPa]	SD [MPa]	CV [%]	μ [MPa]	SD [MPa]	CV [%]
L_0	9564	1238	12.9	4.55	0.43	9.5
L_90	9803	1243	12.7	4.36	0.87	19.9
LC_0	10879	1386	12.7	4.48	0.65	14.5
LC_90	8450	2433	28.8	3.18	0.43	13.7
C_0	4750	0	0.0	1.84	0.00	0.0

**Table 4:** Cross-sectional area *A* and density ρ for UCT specimens with mean value μ, standard deviation SD and coefficient of variation CV.

Printing Pattern	Cross-Sectional Area *A*			Density ρ		
	μ [mm^2^]	SD [mm^2^]	CV [%]	$\mu \;\left[\frac{kg}{m^{3}}\right]$	SD $\left[\frac{kg}{m^{3}}\right]$	CV [%]
L_l	3008.6	10.9	0.4	2012.1	1.8	0.1
L_s	2969.1	16.3	0.5	2005.9	4.5	0.2
LC_l	2657.5	13.2	0.5	1881.4	17.8	0.9
LC_s	2714.0	16.4	0.6	1847.8	6.0	0.3
C_l	2307.8	25.8	1.1	1829.0	25.6	1.4
C_s	2298.0	37.3	1.6	1834.7	16.6	0.9

**Table 5:** Young’s modulus *E* and compressive strength $f_{c}$ derived from UCT with mean value μ, standard deviation SD and coefficient of variation CV.

Printing Pattern	Young’s Modulus *E*			Compressive Strength $f_{c}$		
	μ [MPa]	SD [MPa]	CV [%]	μ [MPa]	SD [MPa]	CV [%]
L_l	18119	814	4.5	44.15	0.84	1.9
L_s	19785	1503	7.6	43.03	0.49	1.1
LC_l	13096	593	4.5	26.92	5.46	20.3
LC_s	13816	469	3.4	28.16	1.40	5.0
C_l	9771	1787	18.3	11.18	2.82	25.3
C_s	7756	3055	39.4	12.43	2.71	21.8

## Data Availability

The raw data supporting the conclusions of this article will be made available by the authors on request.

## Abbreviations

The following abbreviations are used in this manuscript:

3DCP	3D concrete printing
3PBT	Three-point bending test
C	Crosswise printing pattern
DIC	Digital image correlation
FE	Finite element
l	Long specimen length
L	Lengthwise printing pattern
LC	Length-/crosswise printing pattern
s	Short specimen length
UCT	Uniaxial compression test
